# The costs of schizophrenia and predictors of hospitalisation from the statutory health insurance perspective

**DOI:** 10.1186/2191-1991-2-9

**Published:** 2012-05-04

**Authors:** Jan Zeidler, Lara Slawik, Jochen Fleischmann, Wolfgang Greiner

**Affiliations:** 1 Center for Health Economics, Leibniz University of Hannover, Königsworther Platz 1, D-30167, Hannover, Germany; 2 Health Economics, Janssen-Cilag GmbH, Neuss, Germany; 3 Department of Health Economics and Health Care Management, University of Bielefeld, Bielefeld, Germany

**Keywords:** Schizophrenia, Claims data, Hospitalisation, Costs, Predictors, Germany

## Abstract

**Background:**

The aim of the study was to determine the costs of treating schizophrenia from the perspective of the statutory health insurance, as well as the identification of predictors of hospitalisation of formerly stable schizophrenia patients.

**Methods:**

Claims data for the years 2004–2006 were analysed. Patients who did not have to be treated in a hospital as a result of an ICD diagnosis F20 both in the year 2005 as well as also in 2006 were defined as stable patients. In contrast, those patients who had to be treated in a hospital in 2006 because of a diagnosis of schizophrenia were defined as unstable. In addition to the overall healthcare costs, the costs specific to schizophrenia were also analysed. Also, based on binary logistic regression analysis, predictors for hospital treatment were determined.

**Results:**

8497 stable and 1449 unstable patients were identified. The schizophrenia specific costs for stable patients were € 1605 and the overall costs were € 4029 in 2006, respectively. Unstable patients had indication-specific costs amounting to € 12864 and overall health care costs of € 16824. For unstable patients, the costs of hospital treatment were identified as being a substantial cost area. Predictors for a higher probability of hospital treatment were: female patients, at least one rehabilitation measure, at least one stay in hospital in 2004, and being co-morbid with substance abuse. In contrast, older patients, who were treated with concomitant medications, and if they received a continuous drug therapy in all quarters of a year had a lower probability of hospitalisation. In addition, an increased number of visits to a doctor reduced the probability of hospitalisation. The variable ‘depot medication’ were close to significance and the variable ‘inability to work lasting more than six weeks’ had, in contrast, no significant influence.

**Conclusions:**

The schizophrenia specific and overall health care costs of unstable patients were clearly higher than was the case with stable patients and mainly determined by inpatient hospital treatment. A range of potential predicting factors which can be extracted from routine claims data have a positive or negative influence on the probability of treatment in hospital.

## Background

Schizophrenia is a serious illness with considerable economic importance [1]. Because of the specific characteristics of the illness, schizophrenia is considered to be one of the most costly mental disorders [2]. According to official cost data compiled by the Federal Office of Statistics schizophrenia (ICD F20) resulted in direct costs of around 2.0 billion Euro to the statutory health insurance in 2008 [3]. In addition to this, there are much higher indirect costs which, at present, are not recorded or evaluated in any routine statistics [4-7]. The official statistics for 2008 indicate, however, that as a result of this disease in total 86,000 years of employment were lost [3]. The causes of these higher costs lie in the young age, at which patients first become ill (between the ages of 18 and 35), as well as the tendency for the illness to become chronic [8].

Despite the increasing economic importance of schizophrenia, systematic cost analyses based on the use of health economic methods have, up until now, only been carried out sporadically in Germany [7,9,10]. In view of scarce resources in the healthcare system, however, detailed cost analyses are becoming of increasing practical relevance. Gaining precise knowledge of individual cost elements is likewise important, just as is information about the individual and structural causes of variance in claims for benefits [11].

Studies have identified, in particular, hospital stays as being the cost driver [2,5,7,11-14]. Frequent inpatient stays can endanger social and occupational integration, as a result of the associated loss of the ability to work and social participation and limit the individual´s quality of life. Effective healthcare management can contribute to identifying an increased risk of hospitalisation [15-17]. In this way, the medical care of patients could be supported and more efficiently designed using innovative outpatient treatment options such as psycho-education, specialist nursing care, sociotherapy, treatment provided by visiting the patient at home and care provided by caregivers. In order to ensure this, the identification of those factors influencing an increased risk of hospitalisation would be helpful.

Therefore, in the study presented here, not only the costs of treating schizophrenia using a bottom-up approach from the health insurance perspective was undertaken, but also predictors for inpatient hospital treatment of formerly stable patients were identified.

## Methods

### Data basis and study population

Routine data from a large statutory health insurance scheme (which had approx. 6 million insured persons in 2006) for the years 2004–2006 were made available for the study in an anonymous form. The study population included all insured persons who in 2006 had at least one schizophrenia-relevant diagnosis (ICD-10 code F20) in a hospital or at least one verified schizophrenia-relevant diagnosis in two different quarters of the year coded in ambulatory treatment by a specialist.

The basis for the cost and predictor analysis presented here was stable schizophrenic patients, i.e. those persons with schizophrenia who, over a period of two years, did not have to undergo inpatient treatment. Patients were defined as being stable if they did not have to be treated in a hospital in both 2005 as well as in 2006 with an ICD diagnosis F20. In a second step stable patients were compared with unstable patients. Patients were defined as unstable if they were hospitalised because of a diagnosis of schizophrenia in 2006.

However, only those stable and unstable patients were considered in the study who were at least 18 years of age. In addition, only those individuals were considered who were continuously insured with the health insurance throughout the period 2004–2006.

The following data was available on a patient by patient level: socio-demographic data (e.g. age and gender), data on outpatient diagnosis and provided services, hospital stays and treatments, rehabilitation measures, drug prescriptions, remedies, and data relating to sick leave payments. Health services which could not be included explicitly in one of the specific cost domains were summarised in the category ‘other services’. In addition to the resources used that related directly to schizophrenia, it was also possible to depict co-morbidity data as well as costs associated with that.

### Cost calculation and identification of indication-specific resource use

The cost calculation was undertaken from the health insurance perspective. Consequently, indirect costs and costs that arose within other sectors of social insurance were not taken into account. Co-payments and out-of-pocket payments are also not relevant from the perspective of the health insurance as they do not have an impact on their budget [18].

To identify schizophrenia specific drugs, the official German ATC-classification was used. Antipsychotics listed under N05A were defined as relevant substances for the treatment of schizophrenia. For hospital treatments, rehabilitation measures and data related to the inability to work as well as sick leave payments, all procedures that refer back to the ICD diagnosis F20 were classified as being relevant to schizophrenia.

In the area of outpatient medical care the diagnosis and accounting data were only available in separate databanks. Diagnoses in German claims data are generally documented only on a quarterly basis whereas, in contrast, the services provided are documented precisely on a day-by-day basis. Therefore, in a first step, for each insured person those accounting codes (EBM codes) were identified for which, at the same quarter, a schizophrenia-relevant ICD diagnosis F20 was documented. Those accounting codes associated with a diagnosis of schizophrenia in the respective quarter were then adjusted on the basis of the valid German uniform valuation standard (Gebührenordnung Einheitlicher Bewertungsmaßstab: EBM).[19] Specific accounting codes which could be invoiced by psychiatrists, other specialists in nervous disorders and neurologists were defined as indication-specific. This procedure has the consequence that costs of outpatient treatment of schizophrenia that incurred through general practitioner care could not be recorded in relation to the indication-specific resource use. However, these are reported with the cross-indication total costs.

For costing the resource uses official German tariffs were applied in the respective format which was used in the given year. Were prices were available the actual amount paid by the health insurance was included in the analyses. For ambulatory services a point value for services provided according to the Uniform Valuation Scheme (EBM) of 0.035 € was applied [20,21].

Moreover, the use of resources for treatment in the outpatients department of psychiatric institutes was analysed. Treatment in the outpatients department of a psychiatric institute were, in this context, defined as indication-specific and attributed to the costs for outpatient services.

### Study design predictor analysis

In addition to the cost analysis, based on a binary logistic regression model, predictors for probability of inpatient hospital treatment were identified. As predictors for hospital stays, the variables age and gender of the insured person, the type of drug therapy, the use of depot medication, the continuity of drug treatment, the number of visits to a doctor, the use of rehabilitation measures, inability to work for more than six weeks, a stay in hospital as a result of an ICD diagnose F20 in the year 2004 and the presence of a comorbidity “Mental and behavioural disorders as a result of psychotropic substances” (ICD diagnosis F10-F19) were included.

Information regarding the gender of the insured persons could be obtained directly from the data set. Age was taken as an additional variable from the reference data. For the analysis of the influence of the type of drug therapy, the subjects were allocated to one of seven subgroups, depending on the type of medication, using an algorithm proposed by Stargardt et al. [22]. Allocation to the various drug groups was based on the ATC code. Accordingly, patients who had received at least a prescription of the active substance ziprasidone, clozapine, olanzapine, quetiapine, amisulpride, risperidone, zotepine or aripiprazole were allocated to the group of atypical antipsychotics. Within this group of atypical antipsychotics, persons who only received one atypical substance were defined as non-switchers and individuals who had had different atypical substances, either overlapping or one after the other, were defined as switchers.

For first generation antipsychotics, a differentiation into high potency and low potency active ingredients was made. Amongst the high potency antipsychotics, the active ingredients fluphenazine, perphenazine, haloperidol, bromperidol, benperidol, flupentixol, zuclopenthixol, fluspirilene and pimozide were included. The active substances chlorpromazine, levomepromazine, promazine, perazine, thoiridazine, melperone, pipamperone, chlorprothixene, sulpiride and prothipendyl were allocated to the low potency antipsychotics group. Insured persons who only received one antipsychotic were allocated to the non-switchers while others receiving therapy with different antipsychotics were allocated to the switchers.

Insured persons who received both atypical as well as high potency typical antipsychotics were allocated to the group “typical and atypical antipsychotics”. Individuals who received atypical antipsychotics as well as low potency typical antipsychotics were allocated to the group “atypical antipsychotics with adjuvant therapy”. Persons who received atypical antipsychotics as well as high potency and low potency antipsychotics were allocated to the group “typical and atypical antipsychotics”. Finally, subjects who had not been prescribed antipsychotics by the outpatient sector were subsumed in the group “no relevant outpatient drug prescriptions”. The various drug groups were coded as categorical variables. At the same time, as a reference category, the drug group “no relevant outpatient drug prescription” was selected.

With reference to the influence of the drug therapy, an additional analysis was carried out as to whether individuals received drug therapy with depot neuroleptics. Treatment with depot neuroleptics was coded as a binary variable with two categories based on whether a depot prescription has been made or not. As both atypical and typical antipsychotics are used in Germany in depot form for the treatment of schizophrenia, this variable was included in the analysis as an independent predictor.

The continuity of drug treatment as a predictor was analysed by looking at the number of prescriptions in a quarter. When an individual received at least one antipsychotic prescription per quarter, he or she was categorized as being continuously treated.

The number of outpatient treatment procedures was deduced from the number of physician visits. As a further predictor for hospitalisation, the influence of preceding hospital stays was investigated. All those individuals were identified who had already received hospital treatment in the year 2004 with an F20 diagnosis. In addition, an analysis was carried out of whether an individual received a rehabilitation measure because of schizophrenia. Also, all cases were investigated where there was inability to work for more than six weeks because of schizophrenia.

Co-morbidities can also have an influence on the course of the illness of schizophrenic patients. Consequently, patients with co-morbid substance abuse have a higher rate of re-hospitalisation, reduced psychosocial functions, less patient concordance with treatment, as well as a higher relapse rate than patients without such substance consumption [23]. Therefore, all individuals were identified in the data set who, in the outpatient or inpatient sector, had at least one diagnosis of F10-F19.

The predictors were identified for the stable and unstable patients over an observation period of twelve months. The twelve month period used for stable patients was the calendar year 2006. The period used for identifying the predictors individually for unstable patients was the twelve months before the first hospital referral in 2006. A p-value of <0.05 was considered significant.

### Software

Microsoft Access was used for data storage. For data analysis, both SPSS for Windows version 17, as well as Microsoft Excel and Access (Versions 2007), were used.

## Results

In total, n = 8497 stable (85.4%) and n = 1449 unstable (14.6%) patients aged ≥18 years were identified. The mean (SD) age of the stable patients was 49 (±13.3) years and the percentage of female patients was 48.8% (Table 1). The mean (SD) age of the unstable patient subgroup was, 42 (±13,0) years old and the percentage of female patients was 47.8%.

**Table 1:** Characteristics of stable and unstable patients

	Stable patients* (n = 8497)	Unstable patients** (n = 1449)
Age (years), mean (SD)	49 (13.3)	42 (13.0)
Female, n (%)	4118 (48.8)	692 (47.8)
Patients with depot neuroleptics, n (%)	1127 (13.3)	187 (12.9)
Continuously drug treated patients, n (%)	4651 (54.7)	505 (34.9)
Mean number of doctor visits, (SD)	36 (27.7)	29 (31.5)
Number of patients with rehabilitation paid by statutory health insurance, n (%)	22 (0.3)	14 (1.0)
Number of patients with an inability to work episode of more than six weeks, n (%)	289 (3.4)	74 (5.1)
Number of patients with hospital treatment in 2004, n (%)	822 (9.7)	271 (18.7)
Number of patients with co-morbid substance abuse, n (%)	1167 (13.7)	269 (18.6)

* Stable patients were defined as those persons, who did not have to be treated in a hospital in both 2005 as well as in 2006 due to a diagnosis of schizophrenia (ICD F20).

** Patients were defined as unstable if they were hospitalised because of a diagnosis of schizophrenia in 2006.

The schizophrenia specific costs of the stable patients were € 1605 in 2006 (Table 2). Total costs of these patients which also included the costs for co-morbidities were € 4029. The schizophrenia specific costs of the unstable patients (who were treated in a hospital in 2006) are with € 12864 higher on average. The total costs of these individuals were likewise high amounting to € 16824. Schizophrenia specific costs of unstable patients were eight times and overall health care costs were four times higher than for the stable patients, respectively. The percentage of hospital costs specific to schizophrenia was 84.1%.

**Table 2:** Mean costs of stable and unstable patients in the year 2006 in €

	Stable patients* (n=8497)			Unstable patients** (n=1449)		
	**Mean**	**Standard deviation**	**95%-confidence-interval**	**Mean**	**Standard deviation**	**95%-confidence-interval**
Schizophrenia specific costs						
Medication	1124	1394	(1095,1154)	1292	1415	(1219,1365)
Outpatient						
services	327	407	(318,336)	346	433	(323,368)
Remedies	20	194	(16,24)	16	149	(8,23)
Sick leave						
payments	116	1561	(83,149)	331	2020	(227,435)
Hospitalisation	0	0	(0,0)	10816	10000	(10301,11332)
Rehabilitation	2	91	(0,4)	55	1178	(−5,116)
Other services	16	275	(10,22)	8	147	(1,16)
**Total costs**	**1605**	**2266**	**(1557,1653)**	**12864**	**10614**	**(12317,13411)**
Overall costs						
Medications	1554	1940	(1513,1596)	1552	1756	(1462,1643)
Outpatient						
services	726	710	(711,741)	769	1145	(710,828)
Remedies	80	403	(72,89)	48	233	(36,60)
Sick leave						
payments	275	2347	(225,325)	751	3242	(584,918)
Hospitalisations	1233	4567	(1136,1330)	13423	11489	(12831,14016)
Rehabilitation	38	624	(24,51)	120	1446	(45,194)
Other services	123	841	(105,141)	161	977	(111,212)
**Total costs**	**4029**	**6199**	**(3898,4161)**	**16824**	**12653**	**(16172,17476)**

* Stable patients were defined as those persons, who did not have to be treated in a hospital in both 2005 as well as in 2006 due to a diagnosis of schizophrenia (ICD F20).

** Patients were defined as unstable if they were hospitalised because of a diagnosis of schizophrenia in 2006.

**Figure 1:**
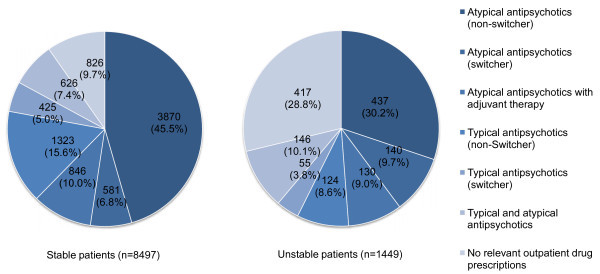
Number of stable and unstable patients in different drug groups.

The different drug groups which the stable patients belonged to in 2006 are illustrated in Figure 1. A total of n = 3870 patients (45.5%) were treated exclusively with an atypical antipsychotic, n = 1323 insured persons (15.6%) were treated with a typical antipsychotic drug only, and n = 826 patients received no drug prescription (9.7%).

The drug groups of the unstable patients were determined for the twelve months before the first hospital referral in 2006. A total of n = 437 patients (30.2%) received only atypical antipsychotic drugs and did not switch to any other active substances during the observation period, n = 124 patients (8.6%) were treated exclusively with a typical antipsychotic drug and the proportion of patients not treated with drugs was n = 417 persons (28.8%) which is higher than for stable patients.

If the results of the logistic regression model are considered, it appears that, apart from the existence of a depot medication, as well as an inability to work for more than six weeks, all independent variables show a significant influence (Table 3). Based on the sign of the logistic regression coefficient (log odds ratio), it can be determined whether the relationship between the dependent variable hospitalisation and the independent variables is positive or negative. These results revealed that patients who were female, had at least one of the rehabilitation measures, at least one hospital stay in 2004 or being co-morbid with substance abuse have a higher probability of hospitalisation. For example, patients with at least one hospital stay in 2004 have a twice as high probability to be hospitalised than patients without a hospital stay in 2004. Older patients, patients who were treated with concomitant medication and patients who received continuous drug therapy in all quarters of a year had a lower probability of hospitalisation. Also, an increased number of physician visits reduces the probability of hospitalisation.

**Table 3:** Results of the logistic regression model for predictors of hospitalisation

**Variable**	**Regression Coefficient(Log Odds Ratio)**	**Standard Error**	**Odds Ratio**	**95%-Odds Ratio Confidence-Interval**	**p-value**
Age	−0.038	0.003	0.962	(0.958,0.967)	<0.001
Gender					
Male					
Female	0.179	0.062	1.196	1.058-1.351	0.004
Drug groups					
No relevant outpatient drug prescriptions					
Atypical antipsychotics (non-switcher)	−1.362	0.088	0.256	(0.216,0.305)	<0.001
Atypical antipsychotics (switcher)	−0.645	0.126	0.524	0.409-0.672	<0.001
Atypical antipsychotics with adjuvant therapy	−0.839	0.126	0.432	0.337-0.553	<0.001
Typical antipsychotics (non-Switcher)	−1.366	0.125	0.255	0.200-0.326	<0.001
Typical antipsychotics (switcher)	−0.762	0.175	0.467	0.331-0.657	<0.001
Typical and atypical antipsychotics	−0.376	0.136	0.686	0.526-0.896	0.006
Depot neuroleptics	0.198	0.105	1.219	0.993-1.497	0.058
Continuously drug treatment	−0.657	0.073	0.519	0.450-0.598	<0.001
Number of doctor visits	−0.008	0.001	0.992	0.990-0.995	<0.001
Rehabilitation	0.734	0.372	2.084	1.006-4.318	0.048
Inability to work episode of more than six weeks	0.200	0.146	1.221	0.917-1.627	0.172
Hospital treatment in 2004	0.739	0.084	2.093	1.777-2.466	<0.001
Co-morbid substance abuse (ICD F10-F19)	0.278	0.081	1.320	1.125-1.548	0.001

The logistic regression model produced a nagelkerke R^2^ of 16.6%. Nagelkerke R^2^ is an pseudo R^2^ which summarizes how much of the variability of the depended variable is successfully explained by the independent variables. As an alternative to the regression model presented above, we also calculated gradual models. For this procedure, the independent variables are not incorporated in the model all at once, but gradually one by one. In summary, the results of the gradual model approach were the same as with the regression model.

## Discussion

On the basis of the results presented in this study, it is possible to make precise statements about the distribution of costs for stable and unstable patients over the various cost domains paid by health insurances. The most significant cost driver was costs for inpatient hospital treatment. With € 10816, these constitute 84.1% of the indication-specific treatment costs of unstable patients.

The results of the logistic regression model have confirmed that there is a significant influence for almost all of the selected predictors on probability of hospitalisation. Only the independent variable ‘depot medication’ was close to significance and ‘inability to work for more than six weeks’ had no significant influence. Patients who were female, had at least one of the rehabilitation measures, at least one hospital stay in 2004 or being co-morbid with substance abuse have a higher probability of hospitalisation than patients in whom these characteristics are not present. In contrast, older patients, who were treated with concomitant medication, and if they received continuous drug therapy in all quarters of a year had a lower probability of hospitalisation. In addition, an increased number of physician visits reduces the probability of hospitalisation.

Other analyses have been done in the past which have investigated the relationship between different influencing factors and inpatient treatment procedures in patients suffering from schizophrenia. However, many studies analyse predictors for rehospitalisation, i. e. the renewed hospital referral of patients who, during the observation period, have already received inpatient treatment at least once [24-29]. Other studies have, at the same time, considered a number of psychological disorders without separately reporting the results of the regression model for schizophrenia patients [30-33]. The diagnosis of schizophrenia was used in these studies as an independent predictor. One study which was concerned with the prediction of relapses and also took into consideration stable patients, used a more comprehensive definition of relapses which, in addition to referrals to an inpatients department of a hospital, also included attempted suicides [34]. Moreover, none of the studies referred to relate to the German care provision context. However, the studies do suggest important factors affecting inpatient treatment procedures. The present study analysed these factors for the first time for the German health care system.

The advantages of this study are that it addresses a large country-wide study population, as well as routine data-supported depiction of clinical daily life. It is clear that Statutory Health Insurance [GKV] routine data has a substantial advantage for health economic cost analyses from the perspective of a health insurance. In addition, in the predictor analysis, a range of variables could be identified that showed a significant influence on the hospitalisation of previously stable patients. Identified predictors are routinely recorded by the health insurance companies and are, in principle, available for use at any time.

In addition to the advantages referred to, the study does, however, also show limitations. The reference data contains not more detailed socioeconomic information, for example regarding income classes or the social status of the individuals. Also, clinical data, such as the severity of the illness, are not available to health insurance companies in Germany in their routine data as regulated by social law. As a result, the two patient groups – the stable and the unstable patients – can only be compared to a limited extent, as differences with regard to the severity of the psychological illness cannot be ruled out. In addition, drugs prescribed in a hospital are included directly in payments made direct to hospitals and are not listed separately in the accounting data of the health insurance. However, inclusion of these costs would only widen the difference in medication costs for these two groups making the unstable group even more costly.

Determination of the total direct costs of psychological disorders is made more difficult by the strong fragmentation of the German care system [35]. Thus, for example, not all medical rehabilitation measures which are made use of because of a diagnosis of schizophrenia are financed by the health insurance companies [36]. This is linked to the division of responsibilities between those who bear the costs of rehabilitation. In Germany, the health insurance companies are traditionally responsible for the rehabilitation of those who are not employed. For the rehabilitation of those in employment or those who are basically capable of working, as a rule, the Statutory Pension Insurance is responsible. The aim of the study presented here was, however, precisely the determination of the use of resources that are relevant from the perspective of a health insurance.

A challenge was presented by the fact that the ICD diagnoses from outpatient care are reported quarterly, whereas the cleared EBM codes, in contrast, are reported on a daily basis. A direct allocation of individual clearing figures to the diagnoses justifying the treatment was therefore not possible. However, with the procedure which we used, it was possible to identify the indication-specific treatment costs generated by psychiatrists, specialists in nervous disorders and neurologists. Treatment provided by general practitioners had, in contrast, to be listed under the overall health care costs.

As the treatment of patients with schizophrenia is partly carried out by general practitioners in Germany, the total costs incurred for the treatment of this illness should be located between the indication-specific and overall costs. Even indication-specific allocation of healthcare services, such as occupational therapy and stress testing or providing support to the patient by home help was not possible in every case. In such cases the resource use had therefore to be included only in the overall health care costs. Accordingly, the schizophrenia specific costs quite definitely reflect the use of resources attributable directly to the treatment of schizophrenia. Moreover, the overall costs also include the use of resources attributable indirectly to the treatment of schizophrenia which cannot be clearly allocated to schizophrenia as well as the costs for treating comorbidities.

The results of the study presented here do, however, provide important information about the disease-specific and overall costs of treatment of stable and non-stable schizophrenia patients from the perspective of the statutory health insurance. Furthermore, this is the first study that identifies predictors for the hospitalisation of previously stable patients for the German healthcare context based on routine data. In future studies it should be explored how the predictors established in the present analysis can be used in clinical practice to identify patients with an increased risk of hospitalisation.

## Conclusions

This claims data study demonstrates that schizophrenia-specific and overall health care costs of unstable patients are clearly higher than is the case with stable patients and mainly determined by inpatient hospital treatment. A range of potential predicting factors which can be extracted from routine claims data have a positive or negative influence on the probability of treatment in hospital. The predictors established in the present analysis should be used in clinical practice to identify patients with an increased risk of hospitalisation.

## Competing interests

LS is an employee of Janssen-Cilag GmbH.

JF is an employee of Janssen-Cilag GmbH.

## Authors’ contributions

JZ was responsible for the conception and design of the study, performed the statistical data analysis and drafted the manuscript. LS helped to draft the manuscript and revised it critically for important intellectual content. JF helped to draft the manuscript and revised it critically for important intellectual content. WG was involved in the conception and design of the study, reviewed the manuscript and revised it critically for important intellectual content. All authors read and approved the final manuscript.
